# The Nanoparticle-Enabled Success of COVID-19 mRNA Vaccines and the Promise of Microneedle Platforms for Pandemic Vaccine Response

**DOI:** 10.1089/dna.2021.0538

**Published:** 2022-01-12

**Authors:** Senta M. Kapnick

**Affiliations:** Fischell Department of Bioengineering, University of Maryland, College Park, Maryland, USA.

**Keywords:** biomaterials, COVID-19 vaccines, nanoparticles and COVID-19 vaccines, microneedle arrays for vaccination, biomaterials and pandemic readiness

## Abstract

The coronavirus disease 2019 (COVID-19) public health crisis has reached critical mass, but interdisciplinary research efforts have provided the global community with the first effective medical intervention to fight the pandemic—COVID-19 vaccines. Two of the vaccines approved for use in the United States and Europe deliver nucleic acid in the form of mRNA, the success of which would not be possible without biomaterials. Lipid nanoparticle (LNP)-based mRNA vaccines, discussed in this perspective, protect nucleic acids from degradation and deliver cargo directly to the intracellular compartment of cells where it is translated into the antigenic severe acute respiratory syndrome coronavirus 2 (SARS-CoV-2) spike protein that triggers protective immune responses. Despite success of LNP-based mRNA vaccines thus far, the pandemic has highlighted the need for emerging technologies that enable rapid development and increased accessibility to vaccination. Microneedle arrays, also discussed in this study, provide features that could lower barriers to vaccine access in resource-poor regions. The ability to exchange antigens within arrays could also facilitate swift vaccine deployment as public health needs evolve (e.g., in response to SARS-CoV-2 variants or entirely new pathogens). Therefore, the COVID-19 pandemic has spotlighted the readiness and value of biomaterials for the prevention and management of disease outbreaks.

## Introduction

Severe acute respiratory syndrome coronavirus 2 (SARS-CoV-2), the causative agent of coronavirus disease 2019 (COVID-19), was first identified in December 2019. In just <2 years, there have been 250+ million confirmed cases and >5 million deaths worldwide [World Health Organization (WHO), 2012]. Studies examining postacute sequelae of the disease reveal that 20% of hospitalized COVID-19 patients are discharged with a new disability (Briggs and Vassall, 2021), including reduced pulmonary function, fatigue, cardiac complications, and psychiatric difficulties (Huang *et al*., 2021; Taquet *et al*., 2021). These data emphasize the continued need for vaccines that prevent coronavirus infection, as well as the development of promising vaccination strategies that are highly accessible and can be rapidly deployed in the case of future outbreaks.

## mRNA Vaccines Are a Novel Effective Strategy for COVID-19 Vaccination

Vaccination is considered the most effective public health tool for combatting disease-associated mortality and morbidity. The urgency of the COVID-19 pandemic ushered in regulatory approval of a new generation of vaccines, known as nucleic acid (mRNA) vaccines. In 2020, Pfizer-BioNTech and Moderna's COVID-19 vaccines (BNT162B2 and mRNA-1273, respectively) (Polack *et al*., 2020; Baden *et al*., 2021) were the first in the United States to receive Emergency Use Authorization (EUA) from the Food and Drug Administration (FDA). More than 900 million doses have been delivered into arms of individuals worldwide (Mathieu *et al*., 2021). Although their durability is under active investigation, this makes mRNA vaccines one of the most widely disseminated COVID-19 vaccine strategies thus far.

Nucleic acid vaccines differ from live attenuated vaccines that serve as the gold standard for immune correlates (e.g., M-M-R II [Merck]). Instead of delivering viral antigen, they deliver part of the viral genetic code in the form of mRNA or DNA that contains instructions for synthesis of viral protein. Once the target antigen is identified by sequencing the viral genome of interest, nucleic acids are synthetically produced for inclusion in vaccine formulations (Chaudhary *et al*., 2021).

After reaching the intracellular compartment of cells, nucleic acids provide instructions for production of viral protein that elicits protective immune responses. Herein lies a distinct advantage of mRNA-based vaccination—an immune response can be triggered against highly immunogenic proteins in the absence of other viral components. In the case of Pfizer-BioNTech and Moderna's COVID-19 vaccines, the mRNA component encodes a genetic variant of the SARS-CoV-2 spike protein that contains two proline substitutions, K986P and V987P, which stabilize spike conformation (Wrapp *et al*., 2020).

The path to COVID-19 mRNA vaccines drew on several breakthroughs in nucleic acid research that provided solutions for three major hurdles to mRNA vaccination. The first was the inherent immunogenicity of mRNA caused by engagement of danger-associated pattern recognition sensors in cells, known as Toll-like receptors (TLRs) (Karikó *et al*., 2004). This obstacle was surmounted by the discovery that a biochemical modification of the uridine nucleotide component (uridine to 1-methyl pseudouridine) permits mRNA to evade unwanted TLR-mediated inflammatory responses that would preclude it for use in humans for vaccination (Karikó *et al*., 2005, 2008). The pseudouridine modification constitutes the backbone of both Moderna and Pfizer-BioNTech's licensed COVID-19 vaccines.

Although mRNA formulations have been tested in several applications, including viral vaccines (Kramps and Probst, 2013; Alberer *et al*., 2017; Bahl *et al*., 2017; Richner *et al*., 2017), two additional hurdles persisted. The first is that naked mRNA is quickly degraded by extracellular enzymes. The second was the need for mRNA to be delivered to the intracellular compartment of cells where the machinery for translation resides. Ultimately, it was the advent of a biomaterials-based vehicle that facilitated the success of mRNA vaccines for COVID-19.

Biomaterials are natural or synthetic materials engineered to interact with biological systems. By packaging mRNA inside biomaterials, the cargo is protected until delivered to its desired location. Significant progress has been made in biomaterials development for vaccine applications (Yenkoidiok-Douti and Jewell, 2020; Zarubova *et al*., 2021), but lipid nanoparticles (LNPs) have emerged as the front-runner for mRNA vaccine delivery.

## Lipid Nanoparticles Enable Delivery of mRNA, Permitting the Success of COVID-19 mRNA Vaccination

LNPs are biodegradable spherical vesicles composed of lipids assembled that encapsulate cargo of interest. In 1995, Doxil became the first nanosized liposomal product to receive regulatory approval for delivery of doxorubicin, a chemotherapeutic for treatment of ovarian cancer. Although several years of optimization were required, in 2018, Onpattro became the first lipid and nucleic acid (siRNA)-based FDA-approved product for the treatment of transthyretin amyloidosis (Akinc *et al*., 2019).

To date, there are eight FDA-approved lipid-based nanoparticle products for treatment ranging from fungal infection to leukemias (Mitchell *et al*., 2021). Decades of materials science research that facilitated regulatory approval of these products has firmly established favorable safety and efficacy profiles that laid the foundation for the rapid successful clinical translation of LNP-based mRNA COVID-19 vaccinations (Akinc *et al*., 2019; Anselmo and Mitragotri, 2019; Hou *et al*., 2021).

The LNPs utilized by Pfizer-BioNTech and Moderna are ∼80–100 nm in diameter and composed of four components: (1) positively charged ionizable lipids, (2) polyethylene glycol that increases particle persistence by reducing binding of proteins designating them for clearance, (3) stabilizing agents, such as cholesterol, and (4) naturally occurring phospholipids (Ciaramella and Himansu, 2020; Gaviria and Kilic, 2021). The latter (4) promotes binding of nanoparticles to cell surfaces, enabling their uptake. Charged lipids (1) enhance the stability of mRNA cargo. After endocytosis, they also facilitate its release into the cytosol. Together, these LNP components permit mRNA vaccines to overcome the cargo protection and intracellular delivery hurdles discussed earlier.

One initial drawback of Pfizer-BioNTech's vaccine was the low-temperature requirement (−80°C) for long-term storage. This posed logistical hurdles for distribution. In contrast, Moderna's vaccine can be stored at −20°C. The current view is that the susceptibility of naked mRNA to hydrolysis dictates strict cold storage conditions. Although proprietary, one can speculate that specific LNP formulations allow for relaxed storage requirements. For example, if the mRNA is located inside the nanoparticle core together with cationic lipids that enhance its stability (Yanez Arteta *et al*., 2018), the susceptibility of nucleic acids to degradation could be minimized (Schoenmaker *et al*., 2021). In agreement with this, Moderna credits their proprietary “lipid nanoparticle properties and structure” with its enhanced storage capabilities (Chung *et al.*, 2020; Simmons-Duffin, 2021). Thus, Moderna's LNP formulation illustrates a key feature in which tunability enables biomaterials-based delivery platforms to overcome hurdles imposed by biological cargo.

Several LNP-mRNA formulations are now in clinical trials for vaccination against influenza (NCT03076385, NCT03345043), Zika virus (NCT04064905), and Rabies virus (NCT03713086), among others (Hou *et al*., 2021). The wealth of scrutinized data from ongoing COVID-19 vaccine monitoring and new clinical trials will shed light on additional advantages and challenges for the LNP-based vaccination strategy. Armed with this information, next-generation LNP platforms will undoubtedly be introduced to further improve durability and immunological correlates of protection that will enhance biomaterials-enabled outbreak responses.

## Microneedle Arrays Provide an Accessible and Highly Modular Platform for Vaccination Against Current and Emerging Infectious Diseases

The global pandemic has revealed a need for technologies that enable rapid development and increased accessibility of vaccines for COVID-19 and emerging diseases. Although several platforms are under active investigation (Andorko and Jewell, 2017; Super *et al.*, 2021), microneedle arrays (MNAs) are one existing biomaterials-based vaccination strategy whose development has been accelerated by the current pandemic (Kim *et al.*, 2020; O'Shea *et al.*, 2021).

MNAs are small patches containing hundreds of minimally invasive needles 50–900 μm in length capable of being loaded with diverse cargo. This includes but is not limited to, nucleic acids, protein subunit antigens, and nanoparticles encapsulating vaccine antigens. Microneedles are fabricated to form one of three primary structures: hollow, solid, or dissolvable needles. Notable differences among structures include loading methods (e.g., coating vs. encapsulation), loading capacity, and structural stability. Dissolvable microneedles are the current front-runner in design. They are manufactured using biodegradable polymers encapsulating large vaccine doses compared with solid microneedles. Because they are degradable, they offer the additional benefit of preventing inappropriate or accidental reuse (Donnelly *et al*., 2010; Hong *et al*., 2013).

The mechanism through which microneedles induce immune responses takes advantage of the unique immunological niche in the skin. Once applied, patches form microscopic pores that allow diffusion of cargo into the dermal and epidermal layers. In the skin, MNA-derived antigens are engulfed by tissue-resident antigen-presenting cells (APCs) that traffic to lymph nodes to initiate immune responses (Oakes *et al*., 2019; Caudill *et al*., 2021). Given that APCs are enriched in the skin and their efficient targeting is critical to achieve protective immunity, vaccination through the skin is believed to be more efficient than other delivery routes using smaller amounts of antigen (Al-Zahrani *et al*., 2012). This is known as a “dose-sparing benefit” and allows MNAs to overcome the potential hurdle of their relatively small loading capacity.

Dissolvable microneedle patches offer several advantages over traditional vaccine administration routes that make them particularly attractive for increasing the accessibility of vaccines. These include elimination of sharps and no need for reconstitution in the field (O'Shea *et al*., 2021).

Another unique feature of MNA vaccines is that because microneedles do not reach pain receptors in the skin, they provide a virtually pain-free vaccine delivery route (Oakes *et al*., 2019). Of note, MNA vaccines can also be self-administered. In 2017, a study showed for the first time in a human clinical trial that influenza vaccination using a self-administered single-dose MNA was well tolerated, immunogenic, and preferred by trial participants over vaccination through the intramuscular route (Rouphael *et al*., 2017). The latter was a significant finding because self-administration is a feature that could improve vaccine coverage, especially in resource-poor countries where trained medical personnel are limited.

Yet another potential benefit of MNAs for vaccine delivery is their stability under different storage conditions. This makes them relevant for deployment in regions where cold storage facilities are scarce, a logistics hurdle for currently approved COVID-19 vaccines (Menon *et al*., 2021). Initial reports of MNA-based vaccines assembled for delivery of DNA encoding the SARS-CoV-2 spike protein alongside adjuvant indicate that vaccine patches can be stored at room temperature for at least 30 days without sacrificing T cell responses (Yin *et al*., 2021).

These data are supported by literature indicating MNA vaccines developed for influenza exhibit activity after storage at 25°C for up to 24 months, exposure to heat (60°C for 4 months), irradiation, and several freeze–thaw cycles (Mistilis *et al*., 2017). Although promising, storage conditions of each individual MNA vaccine candidates will need validation. For example, it is likely that protein subunit cargo loaded into MNAs will be stable, whereas mRNA cargo will exhibit susceptibility to degradation unless chemistries are considered during fabrication.

Our group and several others have leveraged microneedles as a delivery platform in preclinical studies for diverse applications, including a melanoma cancer vaccine (Zeng *et al*., 2017), malaria (Carey *et al*., 2014; Yenkoidiok-Douti *et al*., 2021), viral infections (Pattani *et al*., 2012; Rouphael *et al*., 2017), and tetanus toxoid (Mistilis *et al*., 2017). Similarly, most MNAs for COVID-19 vaccination are in preclinical phases. Kuwentrai *et al.* tested the immunogenicity of dissolvable MNAs encapsulating the SARS-CoV-2 S1 spike protein subunit containing an immunogenic receptor-binding domain (RBD). Importantly, they benchmarked their MNA vaccine against a subcutaneous bolus injection and demonstrated that mice exhibit comparable T cell responses (Kuwentrai *et al*., 2020).

However, there was variation in antibody titers in response to the MNA vaccine. These data may reflect differences in skin penetration of dissolvable microneedles that can make controlling precise dosing challenging. Additional testing of their MNA platform for delivery of mRNA using a luciferase reporter revealed minimal production of the bioluminescent product *in vivo*. These studies are critically important, as they highlight both the advantages and limitations of current MNAs for vaccination.

Another early study in mice receiving an MNA-based SARS-CoV-2-S1 spike protein subunit vaccine adjuvanted with RS09, a synthetic TLR4 agonist, reported strong IgG responses following a prime/boost strategy (Kim *et al*., 2020). Interestingly, the authors emphasize that their previous efforts developing an MNA-based Middle East Respiratory (MERS)-S1 subunit vaccine permitted the rapid design of the MNA-based SARS-CoV-2 vaccine in the study. Their experience supports the possibility that vaccines against emerging pathogens can be rapidly produced by exchanging antigen on existing platforms. This modularity, combined with advances in additive fabrication (3D printing) techniques, make production of patches as a vaccine platform scalable (Caudill *et al*., 2021).

For example, one can imagine a scenario where empty arrays are fabricated and stockpiled. In response to public health needs, vaccine adjuvants and antigen either in the form of relevant variants or entirely new pathogens, could be loaded into MNAs using preapproved chemistries for assembly. This feature makes MNAs particularly attractive as a biomaterials-based platform technology for pandemic response where rapid rollout of low-cost easily distributed disease interventions is paramount (O'Shea *et al*., 2021).

Through the Biomedical Advanced Research and Development Authority (BARDA), the U.S. government has provided $1.9 million to three groups (UConn, Vaxess Technologies, and Vendari) for the development of microneedle skin patches for preventing COVID-19 [United States Department of Health and Human Services (DHHS), 2019]. No human data from clinical trials are available yet, so it remains to be seen whether scaffold-based COVID-19 vaccinations will translate. Nonetheless, these investments demonstrate the promise of MNAs as a tool for developing globally accessible vaccines (Arya and Prausnitz, 2016).

Even if MNAs do not impact the immediate crisis, research into the utility of this vaccine platform spurred by the COVID-19 pandemic will further development of this biomaterials platform that could be adapted to address emerging public health needs.

## Conclusion

Scientists and engineers took advantage of the features of LNPs to rationally engineer efficacious mRNA vaccines that altered the course of the pandemic. This also represents a monumental achievement for the biomaterials field and the future of biomaterials-based clinical translation. Early preclinical data from the emerging MNA vaccine delivery platform suggests that desirable features such as self-administration and the ability to rapidly exchange antigens relevant for public health crises may address deployment hurdles in resource-poor areas. Together, these creative technological advances in biomaterials-based vaccine research represent a silver lining of the pandemic response.
